# Supplementary material for: Wolbachia Infection through Hybridization to Enhance an Incompatible Insect Technique-Based Suppression of Aedes albopictus in Eastern Spain
Source: Insects. 2024 Mar 20;15(3):206. doi: 10.3390/insects15030206 (PMC10971076; doi:10.3390/insects15030206)
Supplement: Supplementary file 1 [file insects-15-00206-s001.zip › S2 Figure.pdf]

Figure S2

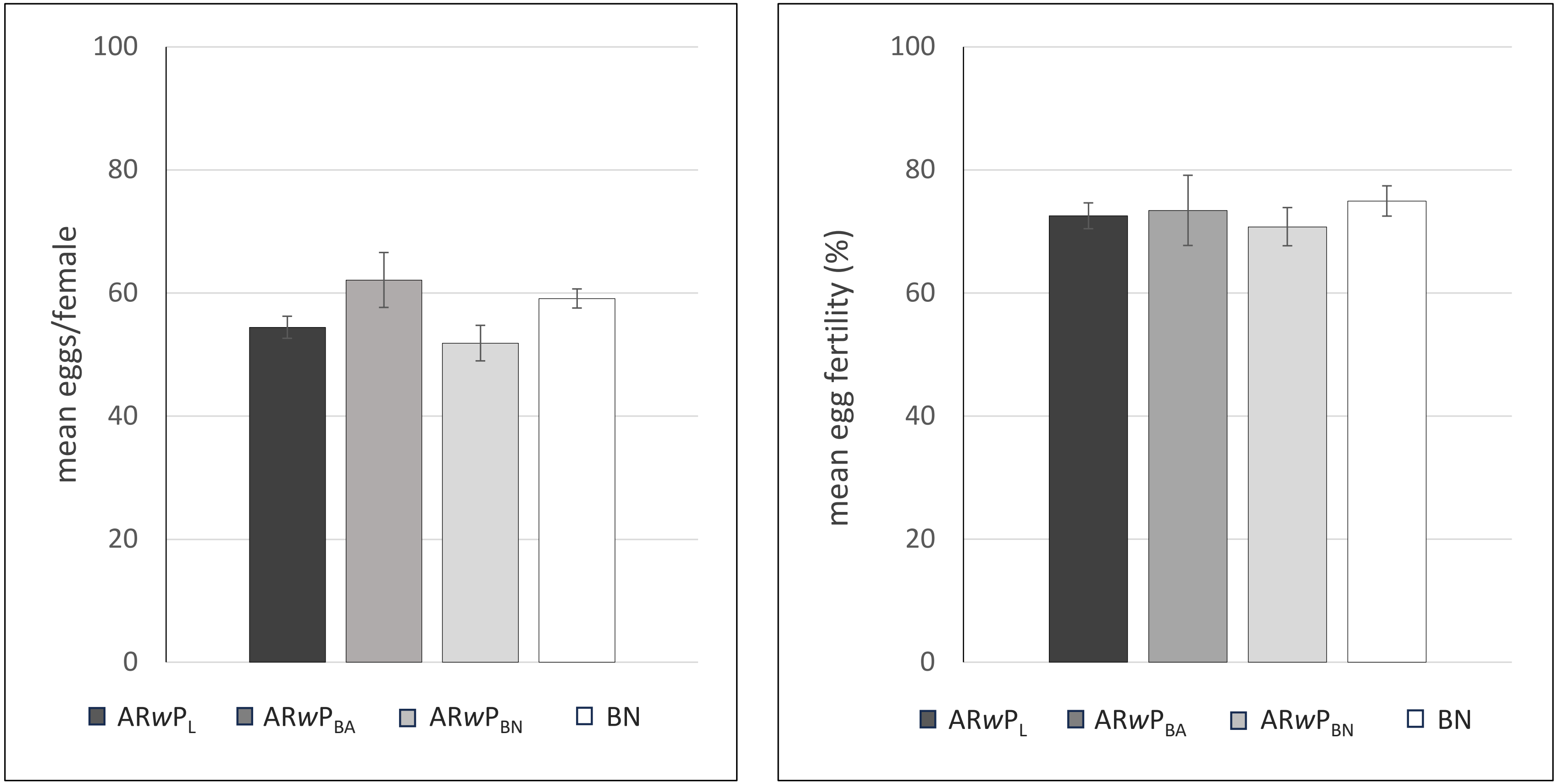

Figure S2. Mean female fecundity (left) and mean egg fertility (right) in *Ae. albopictus* AR*w*P$_L$, AR*w*P$_{BA}$, AR*w*P$_{BN}$, and BN. AR*w*P$_L$: *Wolbachia w*Pip-infected *Ae. albopictus* from Rome (Italy) [31]; AR*w*P$_{BN}$: wild strain of *Ae. albopictus* from Barcelona (Spain) infected with *Wolbachia w*Pip from AR*w*P$_L$ through hybridization; AR*w*P$_{BA}$: aposymbiotic strain of *Ae. albopictus* obtained in Valencia from the wild strain of Barcelona and then infected with *Wolbachia w*Pip from AR*w*P$_L$ through hybridization; BN: wild strain of *Ae. albopictus* from Barcelona (Spain). Error bars show the standard error of the mean of three biological replicates, each containing 19–20 fed females. In both cases, values were not significantly different by One way-ANOVA.
