# Supplementary material for: Wolbachia Infection through Hybridization to Enhance an Incompatible Insect Technique-Based Suppression of Aedes albopictus in Eastern Spain
Source: Insects. 2024 Mar 20;15(3):206. doi: 10.3390/insects15030206 (PMC10971076; doi:10.3390/insects15030206)
Supplement: Supplementary file 1 [file insects-15-00206-s001.zip › S1 figure.pdf]

**Sample**	**GenBank code**	Sex	*Characteristics*
B_N^1	PP212969	♀	Wild-type strain of *Ae. albopictus* from Barcelona (Spain)
B_N^2	PP212970	♀	
B_N^3	PP212971	♀	
B_N^4	PP212972	♀	
B_N^5	PP212973	♀	
B_A^1	PP215893	♀	Aposymbiotic *Ae. albopictus* obtained from the wild-type strain from Barcelona (Spain) by eliminating *Wolbachia*
B_A^2	PP215894	♀	
B_A^3	PP215895	♀	
B_A^4	PP215896	♀	
B_A^5	PP215897	♀	
AR*w*P$_L^1$	PP215898	♂	*Ae. albopictus* infected by transinfection with *w*Pip *Wolbachia* (ENEA, Rome, Italy)
AR*w*P$_L^2$	PP215899	♂	
AR*w*P$_L^3$	PP215900	♂	
AR*w*P$_L^4$	PP215901	♂	
AR*w*P$_L^5$	PP215902	♂	
AR*w*P_B_N^1	PP215903	♂	Wild-type strain of *Ae. albopictus* from Barcelona infected with *w*Pip *Wolbachia* from AR*w*P$_L$ through hybridization
AR*w*P_B_N^2	PP215904	♂	
AR*w*P_B_N^3	PP215905	♂	
AR*w*P_B_N^4	PP215906	♂	
AR*w*P_B_N^5	PP215907	♂	
AR*w*P_B_A^1	PP215908	♂	Aposymbiotic strain of *Ae. albopictus* obtained in Valencia from the wild-type strain of Barcelona and then infected with *w*Pip *Wolbachia* from AR*w*P$_L$ through hybridization
AR*w*P_B_A^2	PP215909	♂	
AR*w*P_B_A^3	PP215910	♂	
AR*w*P_B_A^4	PP215911	♂	
AR*w*P_B_A^5	PP215912	♂	

Figure S1. Genbank codes for the partial COI sequences of the *Aedes albopictus* lines used in this study. No evidence of genetic diversity, regarding haplotypes or even single nucleotides, was detected.
